# Employing Verbal Divergent Thinking to Mitigate Cognitive Decline: Current State of Research and Reasons to Support Its Use

**DOI:** 10.3390/geriatrics9060142

**Published:** 2024-11-03

**Authors:** Vasiliki Folia, Susana Silva

**Affiliations:** 1 Lab of Cognitive Neuroscience, School of Psychology, Aristotle University of Thessaloniki, University Campus, 546 26 Thessaloniki, Greece; 2 Center for Psychology, Faculty of Psychology and Educational Sciences, University of Porto, Rua Alfredo Allen, s/n, 4200-135 Porto, Portugal; susanamsilva@fpce.up.pt

**Keywords:** verbal divergent thinking, creativity, cognitive reserve, dementia prevention

## Abstract

Background/Objectives: Divergent thinking (DT), the ability to generate alternative responses to open-ended problems, has become an increasingly relevant topic in aging research due to its inverse relationship with cognitive decline. Methods: In this narrative review, we explore the latest evidence supporting DT training as a potential strategy for dementia prevention. Results: We identify two pathways through which DT may protect against cognitive decline: (1) by fostering creative cognition and (2) by stimulating DT-related domains. Our findings suggest that verbal DT remains relatively well preserved in older adults, although there is limited empirical evidence to support the idea that DT training enhances creative cognition or DT-related domains in this population. Conclusions: Therefore, while tools designed to enhance DT in older individuals seem promising, it is crucial to rigorously test their effects on the target population to maximize their impact on both the cognitive and psychological domains.

## 1. Introduction

Recently, preventive interventions that enhance cognitive functioning [1] and potentially prevent dementia [2], as opposed to the vast literature on the rehabilitation of dementia, have received increasing attention in scientific research. The operational definition of cognitive stimulation/training distinguishes it from other interventions, such as the broadly termed ‘cognitive intervention’ [3], ‘cognitive enrichment’ [4] and ‘cognitive rehabilitation’, as a preventive method [5,6,7]. Specifically, cognitive stimulation/training involves repeated practice of specific activities using standardized tasks aimed at specific cognitive domains [2]. Recent findings have revealed a strong and positive correlation between cognitive reserve (CR) and divergent thinking (DT) abilities [8,9,10]. Divergent thinking [11] is a classic psychological construct that refers to the ability to generate alternative solutions (“think outside the box”) to an open-ended problem [8,12].

This suggests that leveraging DT in dementia prevention strategies may be beneficial. Due to this established relationship, DT has been proposed as a potential target for cognitive stimulation interventions [13]. Engaging in divergent and creative thinking has been recognized as a method to encourage active aging [14], lower the risk of dementia [9], and decelerate cognitive decline in neurodegenerative diseases [15,16].

In this narrative review article, we synthesize and discuss the current state of evidence on DT as a potential strategy for dementia prevention. Our aim is to provide a conceptual overview of the topic and explore the theoretical frameworks surrounding DT. As research on DT is still emerging and the body of literature remains small, this narrative review seeks to discuss early findings and identify gaps and opportunities within these studies. We focus on interpreting the existing studies, identifying key pathways through which DT may protect against cognitive decline, and highlighting areas in the literature that require further exploration. We examine the rationale for using DT as a dementia prevention strategy by investigating two routes: creative cognition and extended stimulation in areas beyond DT. For the first route, which centers on creative cognition, we discuss the importance of DT, alternative strategies, and compensatory mechanisms. Additionally, we consider two key preconditions for using DT training for cognitive stimulation: the extent to which verbal DT (vDT) is preserved with age and the effectiveness of training programs in producing positive outcomes. In this review, we primarily focus on vDT rather than figural DT (fDT), as the literature suggests that preserved DT abilities, particularly in the verbal domain [17], could be valuable targets for cognitive enhancement programs aimed at promoting active aging and reducing the risk of dementia [9,17,18]. For the second route, we explore whether stimulating DT also benefits other cognitive domains. We review the evidence on whether engaging in DT has broader effects on cognitive functions beyond the immediate impact of DT itself. At the conclusion of this narrative review, we engage in a broader discussion of emerging themes in the field of DT and its application to older adults.

## 2. Divergent Thinking: Definition and Operationalization

### 2.1. Creativity, Divergent, and Convergent Thinking

Thinking divergently is an important component of creativity [19], which is a broader concept [20,21] that also includes convergent thinking (CT), i.e., the ability to select the most appropriate solution to a specific problem [8,21]. While DT involves generating multiple, non-mutually exclusive answers that are functional or appropriate for the situation or request, CT focuses on identifying a single, correct solution [22].

Some authors consider DT to be merely one indicator of creative potential, while others claim that it is the most important indicator [23,24,25]. In some cases, DT is even regarded as synonymous with creativity [26], with results from DT tests often being generalized to reflect creative ability [27,28,29]. In line with [30], we adopt here the narrow definition of DT, i.e., an ability that pertains, but does not equate to, creativity.

### 2.2. Dimensions and Modalities of Divergent Thinking

According to Guilford [11], DT comprises four components: fluency, flexibility, originality, and elaboration. Fluency relates to diversity, reflecting the number of ideas that an individual can generate during brainstorming. Flexibility concerns the ability to use and shift between different perspectives and categories to generate ideas. Originality is measured by the novelty of a solution within a given context and is closely related to infrequent responses to a problem. Finally, elaboration describes the ability to enrich an initial solution with additional details. Later approaches suggest that these components may be dissociated to some extent. For instance, Goff [31] finds evidence that originality may be independent of both fluency and flexibility.

Divergent thinking may be expressed through either vDT or fDT modalities [32,33]. In vDT tasks, individuals are asked to verbalize possible solutions to a problem, such as naming different uses for common objects or predicting the consequences of hypothetical events. Figural DT tasks, on the other hand, primarily involve drawing and elaborating on visuospatial stimuli, as seen for example in tests beyond the Torrance Test of Creative Thinking (TTCT), such as the Pattern Meaning Task [34]. Although both modalities share a common DT component, each modality relies on distinct cognitive abilities and neural resources [35,36,37]. The general neural correlates of DT, as well as those specific to vDT, are discussed further in Section 4.3. Critically, as we will explore in more detail below, aging affects these modalities differently [38,39].

### 2.3. Verbal Divergent Thinking-Related Tasks

To evaluate DT, researchers have developed standardized tasks that incorporate operational definitions of DT. The following are examples of vDT tasks: (1) Alternative Uses (AUs) [22], where participants are asked to list as many diverse uses for everyday objects as they can; (2) Creative Story Generation (CSG) [40], in which participants generate a creative story from sets of three words (e.g., “sleep”, “language”, “sand”); (3) some verbal imagination subscales of the Berliner Intelligenz-Struktur-Test (BIS) [41], where, for instance, participants construct different sentences using given words; (4) The Consequences (Cs) Task [42], which requires test takers to generate ideas related to novel and unconventional situations (e.g., the consequences if people no longer needed or wanted sleep, such as “increased productivity,” “elimination of alarm clocks”, etc.); (5) The Acronyms Task [22], where participants are given acronyms (e.g., SOS) and are asked to list all the terms that could fit into these acronyms (e.g., Substance Owners Society; Suitable Organization of Socks); and (6) The Dissociative Ability Test [43], in which participants generate lists of concepts that are semantically unrelated to a given prompt and to the previously generated word (e.g., summer: “computer, banana, bicycle, ...”).

## 3. Divergent Thinking and Its Relation to Cognitive Reserve

As stated above, DT refers to the ability to think in multiple directions [44,45]. This process involves various cognitive tasks such as association, decomposition, and combination with adjustment [46]. Association refers to the idea that divergent ideas often arise from connecting seemingly unrelated concepts, leading to more unique ideas through remote associations in human memory. The process of decomposition involves breaking down a concept into detailed attributes, fostering original ideas. Combination with adjustment refers to merging elements and altering parts or attributes [43]. Evidence suggests that DT training may help to prevent cognitive decline, as it is linked to cognitive performance. For example, vDT skills in older adults have been associated with scores on the Wechsler Adult Intelligence Scale (WAIS) [10,47,48]. Lately, DT has also been linked to the construct of CR [8,9,10].

About twenty years ago, researchers discovered that some older adults showed no symptoms of disease despite having underlying brain pathology [49]. Concurrently, Stern et al. [50,51,52] introduced the concept of reserve. This model suggests that differences in the neural networks used during tasks account for these disparities, highlighting the ability to employ alternative or flexible strategies to cope with cognitive challenges [50,51,52]. Stern has since proposed a more detailed framework to account for the overarching term of resilience against age- and disease-related changes, encompassing three concepts: CR, brain maintenance (BM), and brain reserve (BR) [53].

The first concept, CR, suggests that diverse life experiences provide a protective buffer against brain damage or age-related decline by employing compensatory processes. CR refers to the adaptability (efficiency, capacity, flexibility) of cognitive processes, which explains why some individuals are more resilient to brain aging and damage. CR-favorable life circumstances include educational level [54,55,56], occupational status and complexity [57,58,59], engagement in leisure activities [10,60,61,62], mental challenges [52,63,64,65,66], social interactions [10,67,68], and personality traits [67,69]. The Cognitive Reserve Index questionnaire (CRI), developed and validated by Nucci et al. [70], is an attempt to gather some of these life circumstances into a single instrument. The second concept, BM, involves reducing age-related brain changes and pathology over time, influenced by genetics and lifestyle. It reflects the idea that the brain is modifiable through experience. The third concept, BR, posits a positive relationship between brain size and the ability to tolerate pathology without clinical symptoms. BR refers to the neurobiological resources available at any given time.

By fostering a flexible and adaptive cognitive style, DT may enhance CR, enabling individuals to better cope with brain changes. Therefore, the relationship between DT abilities and CR lies in the underlying mechanisms that support cognitive flexibility and adaptability. Explicitly, the connection lies in the way that DT supports CR by promoting mental agility through various cognitive processes, such as association and decomposition, which are involved in generating creative and varied ideas and alternative problem-solving strategies. These in turn protect against cognitive decline. Recent studies have shown a strong positive correlation between CR and DT abilities [8,9,10], suggesting that individuals with higher CR might utilize alternative cognitive processes and unconventional brain networks to compensate for pathological conditions [50]. Additionally, there is growing interest in the role of language and semantic tasks as a means to boost healthy cognitive aging, with vDT emerging as a potential proxy indicator of CR [9]. For example, vDT has been found to be linked to a more flexible semantic network [71]. Also, associations between CR, as measured by the CRI (education, leisure, and occupation history) or the Cognitive Reserve Test (CoRe-T), and dimensions of DT,—mostly verbal, have been shown in several studies [8,9,10,47] (Table 1). Moreover, research [8,72] indicates that openness, a personality trait linked to DT [8,46,73], is associated with better cognitive outcomes, such as higher fluid reasoning and vocabulary. These findings suggest, on one hand, that CR might protect vDT [13] and, on the other hand, that vDT enhances CR and sustains active aging [9]. This indicative relationship between CR and vDT has led to the suggestion that vDT could be a target for cognitive stimulation interventions. Exercising vDT could potentially promote mental health by fostering creative cognition in daily life [14], reducing the risk of dementia [9] and slowing cognitive decline during neurodegenerative diseases [15,16]. While some research support this, the body of literature on DT’s effects on aging is still limited compared to studies focused on children.

## 4. First Path Towards Dementia Risk and Prevention: Divergent Thinking as Cognitive Reserve

The concept of creative cognition based on alternative and flexible strategies lies at the core of the CR (functional) construct. Since creativity and flexibility are inherent to DT, the perspective of counteracting cognitive decline by enhancing DT seems promising [13]. However, it remains unclear whether DT (1) simply enhances cognitive performance and/or (2) also plays a role in protecting the brain against disease. To explore both possibilities, researchers must investigate the role of cognitive flexibility in moderating the relationship between brain pathology and cognitive performance.

For step (1), specific requirements must be evaluated based on the available evidence, particularly in the older adult population. Specifically, it is important to determine whether DT is indeed minimally preserved in older adults (see Section 4.1) and whether it can be enhanced through training (see Section 4.2).

For step (2), where it is not yet clear whether DT also plays a role in protecting the brain against diseases, the focus will be on understanding how cognitive flexibility helps individuals maintain cognitive functioning despite brain changes or damage. For example, under assumption (2), we expect that individuals with increased DT would be better able to use alternative strategies when addressing cognitive challenges. This adaptability may enable them to maintain or even improve cognitive functioning despite aging or damage. According to the framework proposed by Stern et al. [53], future research should primarily include longitudinal and neuroimaging studies to investigate how DT predicts cognitive resilience over time, involving both human and animal studies [53]. If cognitive flexibility plays a protective role, one would expect individuals with greater flexibility to show a weaker correlation between brain pathology and cognitive decline, suggesting a higher capacity to adapt to brain changes [53]. Additionally, functional imaging studies should aim to identify specific brain networks that may be more efficient or better support cognitive flexibility in individuals with higher CR. While progress has been made in understanding the neural mechanisms underlying CR, further advancements are still needed [53]. To move in this direction, the neural implementation of DT should be first clarified and thoroughly investigated (see Section 4.3), along with the type of research conducted on DT and neurological diseases (see Section 4.4). We will briefly analyze these sections below.

### 4.1. Verbal Divergent Thinking Is Preserved in Healthy Older Adults

Divergent thinking correlates positively with several factors, such as educational attainment, socioeconomic status [74], and personality traits like openness to experience [8]. Concerning age, DT has been extensively studied in children and young adults due to its relevance in educational settings. In contrast, studies with older individuals are less common and often show mixed results. Early studies evidenced a negative correlation between age and three indices of DT—fluency, flexibility, and originality [27]. In contrast, other studies did not find significant differences in these indices [75,76]. For instance, regarding the fluency index, some studies identified a moderate negative correlation with age [77], a decline in performance among the older adults’ group [78], or a specific decline in the performance of figural tasks [9].

The “peak-decline” hypothesis [79] postulates a peak in DT performance either before [28,80] or during middle age (40–60 years old, approximately) [78,81,82], followed by a decline. However, some have argued that the timed nature of DT tests could introduce a confounding factor, and differences might disappear if timing constraints were removed, workload was reduced, and/or extraneous variables like processing speed or memory skills were controlled for [9,15,75,76,83,84,85]. This aligned with the no-decline hypothesis [86], which even includes findings of improvement with age [84]. Based on the inspection of brain oscillations, Privodnova and Volf [87] proposed that the older individuals may be using different strategies to implement DT, but the task outcomes are nevertheless similar to those of younger participants.

A third hypothesis acknowledges an age-related decline in DT, particularly in vDT, but highlights its nonlinear nature: vDT may peak in middle age and stabilize between the ages of 60 and 99 years old [81,82]. An available explanation for the stabilization of vDT in later decades of life includes a potential shift in cognitive strategy, favoring prior knowledge and semantic elements (DT facilitators) over executive and control components as people age [88], as well as the evidence that verbal abilities remain relatively intact across a human’s lifespan, e.g., [47,88], (see Table 2 for an overview of the theories).

### 4.2. Verbal Divergent Thinking Can Be Enhanced in Children, Adolescents, and Young Adults

Compared to CT, the other crucial component of creativity—DT—appears more challenging to train [89]. According to Cropley [90], this could be because CT relies on recalling knowledge, which tends to be easier than evoking uncommon associations. While both CT and DT involve memory processes, they differ in how this knowledge is utilized. As discussed in Section 2.1, CT refers to the ability to select the most appropriate solution to a given problem by recombining existing knowledge. It involves recalling facts or established solutions and follows a linear, predictable process [90]. In contrast, DT involves forming novel associations between seemingly unrelated ideas, making it a nonlinear and unpredictable process [91]. While convergent tasks typically have concrete methods, divergent tasks depend more on situational factors and broad associations [89]. All the above make DT more difficult to teach directly, as it requires cognitive flexibility and a willingness to think outside traditional frameworks [19,22], both of which are less structured and harder to teach through conventional training methods [89]. Nevertheless, available evidence points to the relevant effects of DT training, i.e., both behavioral and brain-related ones.

Longitudinal studies assessing the behavioral outcomes of DT training have demonstrated DT-enhancing effects in adults (19–35 years old) [92,93,94], adolescents (15–19 years old) [95], and both adolescents (13–16 years old) and adults (23–30 years old) [96] (Table 3).

The CreaTrain program, initially developed by Benedek et al. [92] and Fink et al. [93], has become a significant research tool in this field, with its most notable impact seen in vDT. Regarding brain changes, increases in alpha power [93], along with enhanced activity in creativity-related brain regions, such as the left inferior parietal cortex and the left middle temporal gyrus [97], as well as alterations in resting-state functional connectivity between the medial prefrontal cortex and the middle temporal gyrus [94], have been reported as outcomes of DT training in young adults. According to Haase et al. [89], all dimensions of DT (fluency, originality, flexibility, and elaboration) can be enhanced, although elaboration shows lesser improvement.

Despite these findings, several issues require further research. First, it remains unclear whether and to what extent specific DT training generalizes to other DT tasks or domains. For instance, Baer [98] conducted training with seventh-grade students, focusing on poetry-relevant DT tasks, and found that it significantly improved their poetry-writing skills compared to story-writing. In contrast, Fink et al. [99] investigated fourth graders and showed that vDT training significantly enhances domain-general DT skills. Second, there is evidence that the impact of DT training may depend on individual variables, including brain-related factors. Cousijn et al. [100] studied adolescents and young adults, discovering that variations in DT performance over time were predicted by distinct connectivity patterns that varied among participants. Finally, and most relevant to our focus, research involving older participants appears to be lacking.

**Table 3:** A summary of the studies investigating the effects of DT training on behavioral and brain-related changes in children, adolescents, and young adults. Research in older adults is missing.

Study	Participants (Children, Adolescents, Young Adults)	Type of Training	Key Findings
Fink et al. [99]	Fourth graders	Verbal DT Training	Verbal DT training showed marked effects on domain-general DT skills
Baer [98]	Seventh-grade students	Poetry-relevant DT Training	Greater impact on poetry writing than on other domains, showing domain-specific effects of training
Kleibeuker et al. [95]	Adolescents (15–19 years old)	DT Training (including AUT)	DT-enhancing effects seen in adolescents; changes in DT performance are associated with changes in activation in superior lateral prefrontal cortex activation
Stevenson et al. [96]	Adolescents (13–16) and adults (23–30)	DT Training (several tasks including AUT)	DT-enhancing effects seen in both adolescents and adults
Benedek, et al. [92]; Fink, et al. [93]	Young adults	CreaTrain (vDT) Training	DT-enhancing effects observed in young adults
Fink et al. [94]	Young adults	CreaTrain (vDT) Training	DT training effects on brain activity in the left inferior parietal cortex and left middle temporal gyrus
Wei et al. [97]	Young adults	DT Training (TTCT)	DT training leads to changes in resting-state functional connectivity (medial prefrontal cortex and middle temporal gyrus)
Cousijn et al. [100]	Adolescents and young adults	DT Training (AUT and CAT)	DT training outcomes may depend on individual brain-related factors

Note: DT—Divergent Thinking; AUT—Alternate Uses Task; TTCT—Torrance Test of Creative Thinking; CAT—Creative Ability Test.

### 4.3. Neural Correlates of Divergent Thinking

Divergent thinking seems to involve the activation of multiple neural networks [101] and is primarily regulated by the dorsolateral prefrontal cortex (DLPFC), particularly in aspects of cognitive flexibility (e.g., [102,103]). This makes studies linking the DLPFC to CR especially pertinent. For instance, research using repetitive transcranial magnetic stimulation (rTMS) suggests that older individuals with higher memory performance may recruit the contralateral DLPFC to counteract age-related brain decline, compared to those with lower memory performance [104]. Additionally, research findings from dementia patients [15] support a multi-component neural network that spans the bilateral occipital, parietal, frontal, and temporal lobes, rather than relying on a single brain region or hemisphere [33]. In their meta-analysis, Colangeli et al. [105] investigated brain areas associated with CR proxies in both healthy older adults and Alzheimer’s disease (AD) and MCI patients. Among healthy older adults, the study found that regions such as the DLPFC, anterior cingulate, and precuneus were linked to CR proxies. In contrast, in patients with AD and amnesic-MCI, CR proxies were linked to activation in the anterior cingulate cortex. While Colangeli et al. [105] did not consider DT to be a CR proxy, some of the brain areas that they identified as crucial for CR are also key to DT, such as the DLPFC and the precuneus. Thus, enhancing DT may thus stimulate brain regions linked to CR. Furthermore, the study discussed the hypothesis suggesting the existence of potential compensatory mechanisms in both healthy and pathological aging.

In the broader context of neural correlates of vDT, as previously mentioned in Section 4.2, the behavioral study by Fink et al. [99] found that vDT is enhanced by, and appears to be supported by, domain-general neural networks. This suggests that vDT relies on extensive networks that span multiple cognitive functions (e.g., abstract and associative thinking). Additionally, a few studies have investigated the brain’s structural characteristics related to vDT; however, these studies mainly focused on healthy young adults. Takeuchi et al. [106] employed vDT tasks and found positive correlations between regional gray matter volume (GMV) and vDT in the right DLPFC, bilateral striata, and precuneus. In another study, Vartanian et al. [107] explored the relationship between different scoring methods of the Alternate Uses Task (AUT)—a test of DT—and regional GMV using voxel-based morphometry (VBM). The study found negative correlations between GMV in the left inferior temporal gyrus (ITG) and scores for novelty and usefulness, suggesting a role for the ITG in generating novel and useful ideas. Additionally, EEG studies on creative ideation, specifically DT, have consistently shown right-lateralized brain activity in posterior parietal and occipital regions [108,109].

### 4.4. Divergent Thinking in Patients with Neurological Conditions and Possible Implications for Rehabilitation

To date, few studies have employed standardized tests to assess DT in dementia patients, as stated in [110], and even fewer have focused on the early stages of dementia or different types of dementia and MCIs [16,111]. Overall, these studies typically indicate a decline in DT abilities, emphasizing the importance of early assessment and intervention for patients at risk of dementia (Table 4).

Regarding dementia research, Palmiero et al. [15] reviewed dementia and DT, finding that while dementia generally impairs creativity, individual differences exist. Ross et al. [112] examined creativity changes with age and dementia using tasks like the AUT, among others. The results showed that although older adults scored lower on some measures, most aspects remained stable, even among those with dementia. This suggests that creativity can play a vital role in dementia care and therapy programs aimed at enhancing cognitive functions and well-being. Results on DT in non-AD align with trends observed in AD. Ruggiero et al. [16] studied DT in frontotemporal dementia (FTD) and Parkinson’s disease (PD) using the Divergent Thinking Test (DTT) in visual and verbal domains. FTD patients scored significantly lower than both PD patients and healthy controls, indicating greater creative impairment. Hart and Wade [111] reported vDT deficits in early AD, while Cruz de Souza et al. [113] found that frontotemporal lobar degeneration patients scored lower on both figural and verbal components of the TTCT, with some disinhibited and perseverative responses leading to “pseudo-creative” production in such patients. Similarly, Fusi et al. [18] reviewed studies on DT in FTD, noting that while earlier research suggested that FTD patients may show enhanced creativity, this could stem from an increased drive to produce rather than true creativity. Moreover, they noted that DT abilities, both verbal and figural, are affected differently depending on the FTD subtype. Patients with the behavioral variant can access memories but struggle to recombine them into original ideas due to prefrontal cortex damage. Meanwhile, those with the semantic variant show reduced fluency due to the degradation of semantic memory. There is also one study on MCI and DT, that of Fusi et al. [13], which examined DT focusing on fluency, flexibility, originality, elaboration, and verbal versus figural indicators. The results showed no differences between the MCI and a healthy control group, except for a lower figural score in MCI patients, which improved prediction accuracy by 8%. This suggests that fDT may decline earlier than vDT in MCI, indicating its potential for early diagnosis, while vDT could be a focus for cognitive interventions, as supported by [17,18]. Additionally, recent efforts in neuropsychological rehabilitation aimed at improving cognitive flexibility through programs incorporating both verbal and visual stimuli have been reported [114].

## 5. Second Path to Dementia Risk and Prevention: Shared Resources and Extended Stimulation

As previously discussed, the neural substrates of DT involve multiple brain areas and networks [15,35,109]. This suggests that DT may share neural resources with other cognitive dimensions (Section 5.1), raising the possibility that cognitive improvements acquired through DT training could extend to those dimensions (Section 5.2).

### 5.1. Shared Resources with Memory and Executive Functions

The idea that DT is connected to long-term (both episodic and semantic) memory is logical, as new ideas often arise from the variation in and reorganization of existing knowledge [110]. Nevertheless, experimental stimulation of these two memory systems did not show any effects on DT performance in younger or older participants [115]. On the other hand, a recent study by Zhao et al. [116] found that improvements in working memory updating ability positively influenced performance in both convergent and DT tasks.

Regarding executive functions, they are also engaged in generating new ideas, specifically attention, inhibitory control, and cognitive flexibility [28,117,118,119,120] (see a recent review [121]). In particular, attentional reorientation seems to be especially important for DT [122]. Inhibitory control is crucial for suppressing conventional responses [122], allowing for the emergence of novel ones. Cognitive flexibility enables the transition between various mental frameworks and the application of new criteria to merge disparate ideas [38,123]. In line with this, brain entropy, representing the variability in brain fluctuations, subtending flexibility, has shown positive correlations with DT [124].

### 5.2. Does Divergent Thinking Stimulation Extend to Other Domains?

Very few studies have explored whether DT training can enhance abilities in other domains. Regarding memory, Fink et al. [94] found that DT training impacts brain activity related to semantic memory in university students.

Concerning the potential impact of DT training on executive functions, research is limited, and results are mixed, with most studies excluding older participants. For instance, Vally et al. [125] found no significant effects of DT training on the executive functioning of university students, while Fard et al. [126] reported notable improvements in decision-making, problem-solving, sustained attention, and spatial working memory in very young children (preschoolers) following DT training.

## 6. Discussion

In this review, we examined two key routes related to creative cognition. First, we emphasized the significance of DT, focusing particularly on vDT as a promising and trainable target for cognitive stimulation in aging populations. We explored the extent to which vDT is preserved with age and the effectiveness of training programs in yielding positive outcomes. Second, we evaluated whether stimulating DT enhances other cognitive domains, reviewing evidence of broader cognitive effects. Our findings suggest that vDT can be trained in older adults through standardized methods such as the CreaTraining program. Moreover, vDT appears to be preserved in MCI [13], making it a potentially crucial early assessment and intervention tool for patients at risk of dementia. The potential of DT lies in its ability to generate alternative solutions to open-ended problems, a cognitive skill closely linked to creativity and cognitive flexibility. Verbal DT exercises may play a role in dementia prevention by fostering creative cognition and stimulating DT-related domains. Therefore, promoting vDT could serve as a valuable cognitive exercise for older people.

However, while vDT is promising, the current evidence remains sparce, and more data from older individuals are needed to empirically substantiate its beneficial effects on cognitive decline. There is strong evidence that because vDT is preserved in older people, it allows them to continue contributing to society and potentially enhances their quality of life [76,127]. Nonetheless, although training DT with the aim of transferring benefits to memory and executive functions is a logical step, evidence supporting such a transfer is limited in the former and lacking in the latter, raising questions about the transferability of these benefits in older adults.

The impact of vDT appears to extend beyond cognition, influencing psychological well-being (PWB), which is particularly crucial in later life. Research suggests that DT abilities play a beneficial role in sustaining older adults’ PWB [128]. Current findings indicate that while certain components of PWB, such as coping strategies and emotional competence, decline with age, certain factors can positively influence this relationship. Specifically, DT has an indirect positive effect on PWB by enhancing CR and improving emotional competence in older adults. Future research could inform targeted health interventions and training programs to support the cognitive and emotional skills of older adults, thereby enhancing their overall well-being. Lastly, recent research [110] has explored the relationship between psychological symptoms and DT abilities in an older population, as well as the potential moderating role of CR. Psychological symptoms, such as depression, anxiety, and apathy, were found to negatively impact various DT indices, while CR (measured by educational level) mitigated some of these negative effects [110]. These findings suggest a bidirectional relationship between DT and PWB, implying that creativity, humor, and joy may be “natural partners” that play a bigger role in enriching the aging experience than previously thought.

In conclusion, exploring vDT in older adults may be promising for enhancing both cognitive and psychological well-being in this group. However, several limitations must be considered when interpreting the findings of this narrative review. First, we chose to focus specifically on vDT, thereby excluding potential studies on fDT. While this allowed us to narrow our scope to a particular concept, a future review covering all forms of DT would provide a more comprehensive perspective. Additionally, our focus on dementia and older adults presents certain constraints, as many of the included studies are based on small sample sizes and short follow-up periods, limiting the generalizability of our findings to the broader aging population. Furthermore, there is a need for more robust empirical evidence in this area, and, consequently, a systematic review on the topic would be valuable. However, at the time of conducting this narrative review, such a review was not feasible due to the limited availability of research. Future research should contribute to further investigating DT and its effects on older individuals, which in turn could contribute to a more fulfilling aging experience.

## Figures and Tables

**Table 1:** A summary of the studies examining the relationship between vDT training and CR in older adults.

Study/Author	Focus	Key Findings
Colombo et al. [10]; Colautti et al. [47]; McCrae [48]	Verbal DT skills and WAIS scores	Demonstrated links between vDT skills and WAIS scores, supporting cognitive improvement in older adults
Fusi et al. [13]	CR and vDT	Suggested that CR protects vDT abilities, delaying age-related decline
Colombo et al. [10]; Melendez et al. [8]; Palmiero et al. [9]	CoRe-T; CRI and vDT	Demonstrated that CR (as measured by CoRe-T or CRI) is linked to vDT skills

Note: vDT—verbal Divergent Thinking; CR—Cognitive Reserve; WAIS—Wechsler Adult Intelligence Scale; CoRe-T—Cognitive Reserve Test; CRI—Cognitive Reserve Index questionnaire.

**Table 2:** A summary of studies investigating age-related changes in DT performance, supporting one of the three hypotheses: the negative correlation, peak–decline, or no-decline hypothesis, as well as the nonlinear decline hypothesis (focus on vDT).

	Supporting Hypothesis	Studies
1	Negative correlation between age and DT	Palmiero et al. [9]; Alspaugh et al. [27]; Addis et al. [75]; Roskos-Ewoldsen et al. [76]; Ripple and Jaquish [77]; Jaquish and Ripple [78]
2	Peak–decline hypothesis: DT peaks before middle age and declines afterwards OR No-decline hypothesis: DT does not decline with age and may improve in older individuals or differences may diminish once extraneous variables (e.g., timing, processing speed) are controlled	Ruth and Birren [28]; Jaquish and Ripple [78]; Alspaugh et al. [80]; Palmiero [81]; Reese et al. [82] Palmiero et al. [9]; Palmiero et al. [15]; Addis et al. [75]; Roskos-Ewoldsen et al. [76]; Foos and Boone [83]; Leon et al. [84]; Lindauer [85]; Sasser-Coen [86]; Privodnova and Volf [87]
3	Nonlinear decline in verbal DT (vDT): Stable verbal abilities across age	Colautti et al. [47]; Palmiero [81]; Reese et al. [82]; Park et al. [88]

**Table 4:** A summary of the studies investigating DT in neurological patients with dementia patients, different types of dementia and MCI, and their clinical implications.

Study	Dementia Type	Main Focus	Key Findings	Clinical Implications
Palmiero et al. [15]	Different types of dementia	Review	Creativity declines in dementia, but individual differences exist	Creativity may play a role in therapy, despite overall cognitive decline in dementia
Ross et al. [112]	Aging & dementia	AUT task (among others)	Older adults, including those with dementia, showed stable creativity on AUT, despite age-related declines in other domains	Creativity remains relatively stable with age and dementia, suggesting a role for creativity in enhancing cognitive functions in dementia care
Ruggiero et al. [16]	FTD	DTT (verbal and figural)	Reduction in DT abilities in FTD patients in comparison to healthy controls	Supports early intervention for dementia risk based on cognitive markers
Hart & Wade [111]	Early AD	vDT abilities	Decline in DT abilities	Emphasizes the importance of evaluating DT in the early stages of cognitive decline
Cruz de Souza et al. [113]	Frontotemporal lobar degeneration	TTCT (verbal and figural)	Patients scored lower on the TTCT, often displaying “pseudo-creative” responses	Frontotemporal lobar degeneration patients exhibit disinhibited or perseverative responses, reflecting impaired true creativity
Fusi et al. [13]	MCI	DT skills in MCI patients	No significant differences between MCI and healthy controls except for a lower figural score in MCI	Figural DT could serve as an early marker for cognitive decline in MCI, and vDT could be a target for CR-based training to mitigate dementia risk

Note: DT—Divergent Thinking; vDT—verbal Divergent Thinking; CR—Cognitive Reserve; AUT—Alternate Uses Task; FTD—frontotemporal dementia; DTT—Divergent Thinking Test; AD—Alzheimer’s disease; TTCT—Torrance Test of Creative Thinking; MCI—Mild Cognitive Impairment.

## Data Availability

Not applicable.
